# Anesthesia Management Using Remimazolam for Lower Limb Amputation in a Patient With Septic Shock Due to Necrotizing Fasciitis

**DOI:** 10.7759/cureus.54281

**Published:** 2024-02-16

**Authors:** Haruko Okazaki, Yusuke Ishida, Miki Wada, Reon Kobayashi, Katsunori Oe

**Affiliations:** 1 Anesthesiology, Showa University School of Medicine, Tokyo, JPN; 2 Anesthesiology, Yokohama Asahi Chuo General Hospital, Kanagawa, JPN

**Keywords:** surgery, anesthesia, remimazolam, septic shock, necrotizing fasciitis

## Abstract

We report a case of a patient with necrotizing fasciitis and septic shock caused by streptococcal toxic shock syndrome, who was anesthetized and managed with remimazolam. The patient, a woman in her 40s, was admitted to the ICU with a diagnosis of necrotizing fasciitis of the right lower extremity and septic shock and was scheduled for above-the-knee amputation under general anesthesia. She was anesthetized with remimazolam for sedation and fentanyl and remifentanil for analgesia. Intraoperatively, we were able to maintain hemodynamic stability with similar or only slightly higher doses of circulatory agonists during admission. In the present case, remimazolam, an ultrashort-acting benzodiazepine, was safely used to provide anesthesia to a patient in septic shock due to necrotizing fasciitis, who was receiving high doses of vasopressor agents for cardiovascular support, as it was necessary to select an anesthetic drug that would cause minimal circulatory depression.

## Introduction

Sepsis and septic shock exhibit high mortality rates, requiring rigorous intensive care management and life-saving interventions [1]. In certain instances, there arise circumstances where surgical interventions are required as part of the treatment process to manage the origin of the infection. The anesthesia management of such patients can be challenging, especially considering that the patient might be in a state of shock. Hemodynamic stability might easily be compromised by anesthetics, making careful selection and monitoring of the drug dosage crucial [2]. Streptococcal toxic shock syndrome (STSS) is a severe infectious condition that follows a rapid and potentially fatal course. Characterized by swift progression, it often leads to multiple organ failure, such as acute respiratory distress syndrome, disseminated intravascular coagulation, and renal dysfunction, with a notably high mortality rate [3]. In cases such as the one presented here, where STSS-induced necrotizing fasciitis progressed rapidly, early surgical intervention is crucial [4], often requiring general anesthesia. Remimazolam, which became available in Japan in 2020, is an ultrashort-acting benzodiazepine [5]. Here, we report the successful and safe use of remimazolam for anesthesia management in a patient undergoing above-the-knee amputation who was in a state of septic shock caused by necrotizing fasciitis. Written and informed consent was obtained from the patient for the publication of this case report and all accompanying images.

## Case presentation

**Figure 1:**
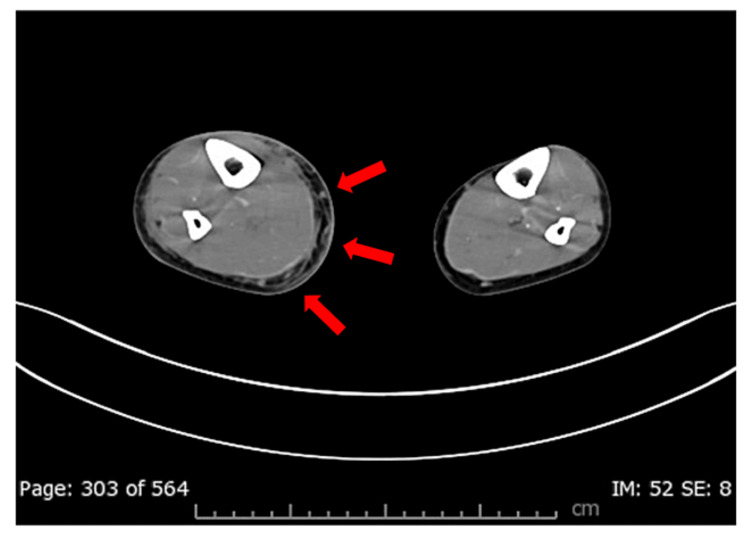
CT scan of the calves A CT scan of the calves showed evidence of swelling and subcutaneous edema in the right lower limb (red arrow).

The patient was a female in her 40s, 161 cm tall, weighing 52.7 kg (American Society of Anesthesiologists-Physical Status 3, ASA-PS 3), who had been under follow-up at our cardiology department for antithrombin III (AT III) deficiency. She presented to our hospital with complaints of swelling and pain in the right lower limb, and further investigation led to a diagnosis of necrotizing fasciitis caused by STSS, for which she was admitted to the ICU. Clinically, redness, blister formation, and blister rupture were observed in the right lower limb. A CT scan revealed swelling and subcutaneous edema in the right lower limb (Figure 1).

**Figure 2:**
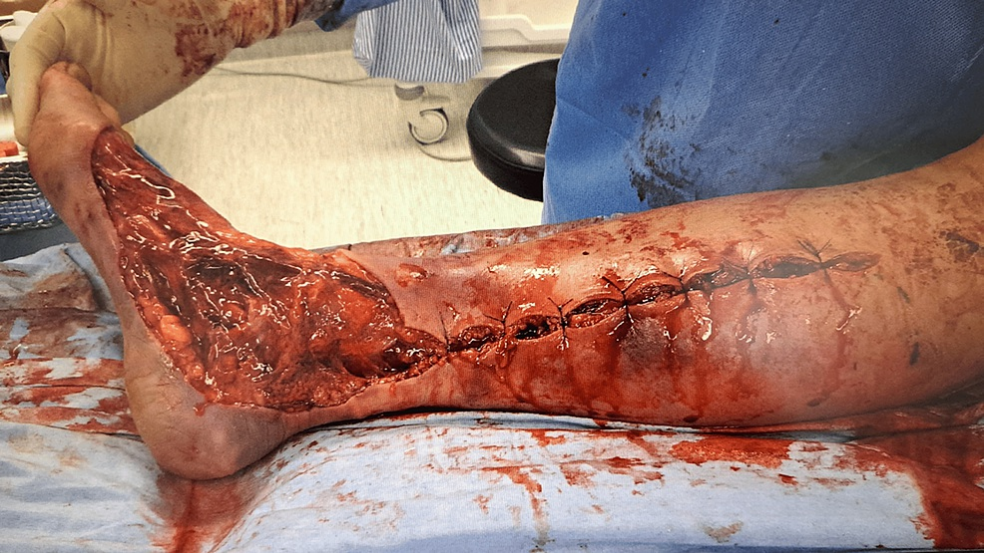
View of the right lower extremity after fasciotomy and drainage Fascia incision and drainage of the right calf were performed for infection control.

Transthoracic echocardiography indicated an ejection fraction of about 50%. No valvular diseases were observed, and there was no evidence of pericardial effusion. Fascia incision and drainage of the right calf were performed for infection control (Figure 2).

However, her general condition gradually worsened, and she was diagnosed with septic shock, for which large amounts of fluids and vasopressors (norepinephrine of 0.3 µg/kg/min) were administered. Furthermore, because of worsening respiratory status, endotracheal intubation and mechanical ventilation were initiated, and renal replacement therapy was initiated with polymyxin B immobilized fiber column direct hemoperfusion because of worsening renal function. However, as there was no improvement in her symptoms, a decision was made to perform amputation of the right lower limb, and anesthesia management was requested. In the context of septic shock, circulation was maintained through the administration of 0.3 µg/kg/min norepinephrine and 0.5 U/h vasopressin, as high-dose circulatory agonist therapy. Given the anticipated risk of a cardiodynamic breakdown, the plan was to manage anesthesia using agents that cause minimal circulatory suppression.

**Figure 3:**
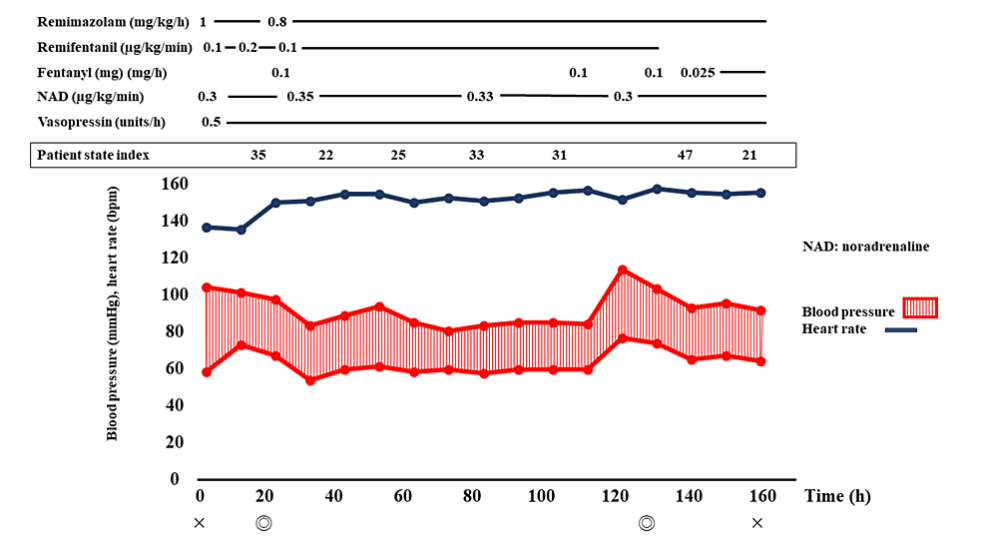
Patient’s anesthesia record PSI values of 25 to 50 are considered the optimal level of sedation for general anesthesia. Her PSI values ranged between 20 and 40.

Anesthesia was induced with 1 mg/kg/h remimazolam, 0.1 µg/kg/min remifentanil, and 50 mg rocuronium and maintained with 0.8-1.0 mg/kg/h remimazolam and 0.1-0.2 µg/kg/min remifentanil, guided by hemodynamic parameters (invasive arterial pressure) and the patient state index (PSI), measured using a Sedline® monitor (Masimo Co., Irvine, CA). Intraoperatively, her PSI values ranged between 20 and 40 (Figure 3).

Additionally, administration of 0.3 µg/kg/min norepinephrine and 0.5 U/h vasopressin was continued from the patient's entry into the operating room. With this management, the surgery was completed successfully without any cardiodynamic collapse. At the end of the surgery, remimazolam was discontinued, and the patient was transferred to the ICU. Anesthesia duration was 2 h and 34 min, surgical duration was 1 h and 43 min, total infusion volume was 1,000 mL, and blood loss volume was 373 + α mL. Blood transfusion comprised six units of packed red blood cells and 10 units of platelets. After entering the ICU with the endotracheal tube in place, the patient was successfully weaned from mechanical ventilation and extubated on the ninth postoperative day. The patient had a favorable recovery and was transferred from the ICU to a general ward on the 23rd postoperative day.

## Discussion

This case involved a patient in a state of septic shock. Upon entry to the operating room, her systolic blood pressure was maintained at approximately 90 mmHg with the use of 0.3 µg/kg/min norepinephrine and 0.5 U/h vasopressin, as high-dose circulatory agonist therapy. Importantly, considering the risk of a cardiodynamic breakdown associated with anesthetic drugs, choosing sedatives with minimal circulatory suppression was imperative for successful anesthesia management.

Remimazolam, which became available in Japan in 2020, is an ultrashort-acting benzodiazepine [5]. Similar to propofol and midazolam, it allows for the assessment of the patient's consciousness level through electroencephalography (EEG) monitoring [6,7]. Additionally, remimazolam has a shorter half-life compared to midazolam, among other distinctive features [5]. Generally, inhalation anesthetics, such as sevoflurane and desflurane, have potent vasodilatory effects. A previous comparative study between inhalation anesthetics and remimazolam in patients undergoing ablation procedures presented a significant need for vasopressors in the group receiving inhalation anesthetics [8]. Furthermore, when choosing total intravenous anesthesia with propofol, it is important to note that propofol has a stronger circulatory suppressive effect compared to remimazolam [9,10]. Therefore, the use of these sedatives might potentially exacerbate the shock state, making their administration challenging. On the other hand, remimazolam has a lower circulatory suppressive effect. Previous reports have suggested that even in cases with compromised cardiac function, the use of remimazolam allowed the maintenance of stable hemodynamics [11,12]. While remimazolam has several advantages compared to other sedatives, being a relatively new drug, it requires careful attention to potential side effects. As of now, a reported notable complication associated with remimazolam is anaphylaxis attributed to the drug [13,14]. Thus, caution regarding the possibility of anaphylactic shock is advised when using remimazolam. Additionally, there have been reports of resistance to remimazolam in patients who have been on long-term benzodiazepine therapy [15]. Therefore, caution is necessary when considering the use of remimazolam in such patients.

In this case, the level of sedation was continuously monitored using PSI, through ongoing assessment of the EEG. Regarding circulation, continuous observation of blood pressure fluctuations was achieved through invasive arterial pressure measurements. This management was successful, with no events suggestive of a cardiodynamic breakdown. There are no previous reports of the use of remimazolam for anesthesia management in patients with septic shock. However, based on our experience with this case, remimazolam might be effective in the anesthesia management of patients with septic shock.

The impact of remimazolam on sepsis has been investigated primarily through experiments targeting septic rat models, as reported by Fang et al. [16]. According to their findings, remimazolam activates peripheral benzodiazepine receptors in a dose-dependent manner, inhibiting macrophage p38 phosphorylation. This action is likely associated with a reduction in the inflammatory response related to sepsis-induced acute hepatic impairment in rats. This animal experiment suggested that remimazolam potentially exerts an anti-inflammatory effect in clinical settings. Further research is required to understand the implications of remimazolam in sepsis and how it might impact patients in clinical situations.

## Conclusions

In conclusion, we were able to conduct successful anesthesia management of a patient with septic shock secondary to necrotizing fasciitis. Despite the challenges involved in managing anesthesia for patients in a state of shock, the use of remimazolam allowed for effective anesthesia management with adequate sedation. Our experience with this case suggests that remimazolam can be safely utilized in the anesthesia management of patients presenting with septic or other forms of shock.
